# Rare occurrence of immunotherapy-related gastritis and duodenitis in a single tertiary medical center: a diagnostic challenge

**DOI:** 10.1093/oncolo/oyaf349

**Published:** 2025-10-14

**Authors:** Tal Etan, Iddo Bar-Yishay, Ariel Greenberg, Eli Brazowsky, Miriam R Brezis, Shlomit Strulov Shachar, Mor Miodovnik, Ido Wolf, Yasmin Leshem

**Affiliations:** Oncology Division, Tel Aviv Sourasky Medical Center, Tel Aviv 6423906, Israel; Gray Faculty of Medicine, Tel Aviv University, Tel Aviv 69978, Israel; Gray Faculty of Medicine, Tel Aviv University, Tel Aviv 69978, Israel; Department Gastroenterology and Hepatology, Tel-Aviv Sourasky Medical Center, Tel Aviv 6423906, Israel; Gray Faculty of Medicine, Tel Aviv University, Tel Aviv 69978, Israel; Department Pathology, Tel-Aviv Sourasky Medical Center, Tel Aviv 6423906, Israel; Gray Faculty of Medicine, Tel Aviv University, Tel Aviv 69978, Israel; Department Pathology, Tel-Aviv Sourasky Medical Center, Tel Aviv 6423906, Israel; Oncology Division, Tel Aviv Sourasky Medical Center, Tel Aviv 6423906, Israel; Gray Faculty of Medicine, Tel Aviv University, Tel Aviv 69978, Israel; Oncology Division, Tel Aviv Sourasky Medical Center, Tel Aviv 6423906, Israel; Gray Faculty of Medicine, Tel Aviv University, Tel Aviv 69978, Israel; Oncology Division, Tel Aviv Sourasky Medical Center, Tel Aviv 6423906, Israel; Gray Faculty of Medicine, Tel Aviv University, Tel Aviv 69978, Israel; Oncology Division, Tel Aviv Sourasky Medical Center, Tel Aviv 6423906, Israel; Gray Faculty of Medicine, Tel Aviv University, Tel Aviv 69978, Israel; Oncology Division, Tel Aviv Sourasky Medical Center, Tel Aviv 6423906, Israel; Gray Faculty of Medicine, Tel Aviv University, Tel Aviv 69978, Israel

**Keywords:** immunotherapy, immune-related adverse events, gastritis, duodenitis, immune-related gastritis or duodenitis, re challenge

## Abstract

**Introduction:**

Immune checkpoint inhibitors (ICI) have revolutionized cancer treatment. While generally well-tolerated, some immune-related adverse events (ir-AEs) can impact patient care, highlighting the need for accurate diagnosis. Immunotherapy-related gastritis and duodenitis (ir-GD) is a rare ir-AE. Due to its low incidence and nonspecific symptoms, standardized diagnostic criteria and evidence-based treatment guidelines are lacking.

**Methods:**

We conducted a retrospective analysis of patients undergoing esophagogastroduodenoscopy (EGD) while receiving ICI. Our diagnostic criteria for ir-GD required at least two of three gastritis or duodenitis (GD) criteria (symptoms, EGD findings, or microscopic findings) along with at least one ICI-related criterion. Patients with ir-GD were compared to those without ir-GD.

**Results:**

Of 2553 patients treated with ICI between 2017 and 2023, 62 (2.4%) underwent EGD, of whom nine (0.4%) met the ir-GD diagnostic criteria. Nine other patients (0.4%) had GD unrelated to ICI. Notably, three of the nine patients (33%) with ir-GD had preexisting inflammatory gastrointestinal conditions, compared to two of 53 patients (4%) in the non-ir-GD cohort (*P* = .019). Patients with ir-GD were significantly more symptomatic (100% vs 58%, *P* = .009). However, no single symptom was specific to ir-GD. While eight of nine (89%) patients with ir-GD were treated with proton pump inhibitors, only three (33%) required corticosteroids. Symptom resolution occurred in all patients, and three patients successfully underwent rechallenge.

**Conclusion:**

ir-GD is a rare ir-AE that can be challenging to distinguish from GD caused by other etiologies. Once diagnosed, clinicians may consider nonsteroidal treatment approaches in mild cases and, in selected cases, even ICI rechallenge.

Implications for practice Immune checkpoint inhibitor (ICI)-related gastritis and/or duodenitis (GD) are two rare immune-related toxicities. Due to their low incidence and nonspecific presentation, standardized diagnostic criteria and evidence-based guidelines are lacking. We investigated the incidence, clinical features, diagnostic workup, and outcomes of immunotherapy-related gastritis or duodenitis (ir-GD). We propose a novel diagnostic approach requiring at least two of three GD criteria and at least one ICI-related criterion, offering a practical and standardized method to facilitate diagnosis. Our findings suggest that ir-GD is a rare but manageable toxicity. Corticosteroids may be avoided in mild cases, and ICI rechallenge can often be performed safely.

## Introduction

Immunotherapy has revolutionized cancer treatment across a wide range of malignancies, significantly prolonging patient survival. Its mechanism of action involves disrupting immune tolerance toward cancer cells by targeting neo antigens arising from tumor-specific mutations.^1^ However, this enhanced immune activation can also disrupt tolerance to self-antigens and lead to immune-related adverse events (ir-AEs).^2^

Gastritis and/or duodenitis (GD), including ulcer-forming GD, is a common disorder in the general population with a prevalence of approximately 0.5%.^3^^,^^4^ Endoscopic appearance can vary, ranging from patchy erythema in mild cases to mucosal erosions or ulcerations in more severe cases. Discordance between endoscopic findings and histology is common, occurring in more than one-third of cases.^5^^,^^6^  *Helicobacter pylori* (HP) and nonsteroidal anti-inflammatory drugs (NSAIDs) are the primary risk factors for GD in the general population.^7^^,^^8^ In cancer patients, additional risk factors, including radiation, chemotherapy, and corticosteroid treatment, can also damage the mucosa.^9^

The occurrence of immune-related gastritis or duodenitis (ir-GD) in patients treated with immune checkpoint inhibitors (ICI) has been reported to range from 0.8% to 1.4%.^10–12^ The current understanding of ir-GD is based on a few case series and retrospective studies that did not systematically exclude other common etiologies leading to GD in cancer patients. The present study aims to explore the incidence and management of this rare toxicity and provide a practical approach to differentiate between ir-GD and non-treatment-related GD in patients receiving ICI.

## Methods

### Study design

This retrospective cohort study was conducted at Tel Aviv Medical Center (TLVMC), a tertiary oncology facility in Israel. To assess the incidence of ir-GD among patients treated with ICI, all patients aged 18 years or older who underwent esophagogastroduodenoscopy (EGD) between 2017 and 2023 while receiving ICI were identified in the electronic medical records, using MDclone software. Patients were included if their EGD was performed at least two weeks following treatment initiation and up to 30 days after their last treatment session. We further contacted medical oncologists at our center to identify additional patients diagnosed with ir-GD whose EGD had been performed outside our facility. Patients were excluded if they had gastric or duodenal malignancies, underwent EGD for percutaneous endoscopic gastrostomy (PEG) tube insertion, or had received only a single dose of immunotherapy. The study was approved by the local ethics committee of TLVMC institutional review board (0618-19 TLV).

### Data collection

The electronic medical records of all patients were reviewed (TE, YL) to collect data regarding demographics, relevant medical history, cancer diagnosis, oncologic treatments (including radiotherapy and systemic therapies), and other immune-related adverse events (ir-AEs). Common Terminology Criteria for Adverse Events (CTCAE) were used for adverse events grading. Additional data were collected on gastrointestinal (GI) symptoms, indications for EGD, and the endoscopic and microscopic EGD findings. Patients undergoing EGD as part of an anemia workup, without gastrointestinal bleeding (GIB), were considered asymptomatic. A gastroenterologist (IBY) reviewed all diagnostic EGDs, and a gastrointestinal pathologist (AG) reviewed all biopsies from available patients with ir-GD. Data lock was set for July 2024. Overall survival (OS) was calculated from the EGD date to the date of last follow-up.

### Ir-GD diagnosis

We propose a novel method for ir-GD diagnosis (Table 1). The method incorporates six diagnostic criteria: three for identifying GD and three for establishing an immune-related etiology. A diagnosis of ir-GD required meeting at least two of the three GD criteria, along with at least one criterion indicating a causal association with ICI therapy. This diagnostic framework is considered appropriate and has been applied in the assessment of various other ir-AEs.^13–15^

**Table 1.** Diagnostic criteria for immune-related gastritis or duodenitis (ir-GD) used in this study.

	Criteria	Inclusion
**Gastritis or duodenitis criteria**	1. Symptoms suggestive of gastritis or duodenitis	Epigastric pain, nausea and vomiting, dysphagia, GIB, dyspepsia
	2. Suggestive findings on EGD	Erosive or erythematous gastritis or duodenitis, ulcers
	3. Histopathological pattern of inflammation	Chronic active gastritis, intraepithelial lymphocytes, atrophic gastritis or duodenitis. Negative HP staining
**Immunotherapy related criteria**	4. No alternative etiology can better explain the findings	Examples for alternative etiologies include HP-induced, drug-induced (NSAIDs, corticosteroids, chemotherapy^a^ etc.), radiation-induced^b^, portal hypertension
	5. Clinical improvement following corticosteroid treatment	
	6. ICI rechallenge resulting in symptom recurrence	
**A diagnosis of ir-GD requires at least two of three gastritis or duodenitis criteria along with at least one immunotherapy-related criterion. All other gastritis and/or duodenitis cases were classified as non-ir-GD.**		

Abbreviations: EGD, esophagogastroduodenoscopy; GIB, gastrointestinal bleeding; HP, *Helicobacter pylori*; ICI, immune checkpoint inhibitors; ir-GD, immune-related gastritis or duodenitis; NSAIDs, nonsteroidal anti-inflammatory drugs.

a recent chemotherapy was defined as receiving the last chemotherapy treatment less than 4 months before the EGD.

b recent radiotherapy to adjacent tissue was defined as radiotherapy to the chest or abdomen in the past 4 months.

### Statistical analysis

Categorical variables were presented as patient counts and percentages, while continuous variables were reported as means and standard deviations. The two-sided Fisher’s exact test was used to compare categorical variables, and the Mann–Whitney *U* test was applied for continuous variables. Statistical analysis was performed using SPSS version 25.

## Results

### Incidence of ir-GD

Between 2017 and 2023, 2553 cancer patients received ICI at our medical center. Of these, 62 eligible patients (2.4%) underwent EGD, including one case performed outside our facility. Using the ir-GD diagnostic criteria outlined in Table 1, nine cases of ir-GD were identified, representing 0.4% of the cohort. An additional 0.4% were diagnosed with GD unrelated to ICI.

### Demographic and clinical characteristics of patients with and without ir-GD

As shown in Table 2, there were no significant differences in age or gender between patients with and without ir-GD (median age: 67 vs 68 years, *P *= .992; female: 33% vs 45%, *P *= .719). The cohort included 28 patients (45%) with non-small cell lung cancer (NSCLC), 7 (11%) with melanoma, 7 (11%) with hepatocellular carcinoma (HCC), and 20 (32%) with other cancer types. Most patients (*n* = 36, 58%) received ICI as first-line therapy for metastatic disease. Most patients (*n* = 43, 70%) received anti–PD-1 therapy, while 12 (19%) received anti–PD-L1 therapy, and 7 (11%) were treated with a combination of ipilimumab and nivolumab. No significant differences were observed in primary tumor origin (*P *= .410), treatment setting (*P *= .228), or type of ICI received (*P *= .511) between patients with and without ir-GD.

**Table 2.** Baseline demographics and clinical characteristics of patients with and without immune-related gastritis or duodenitis (ir-GD).

	Ir-GD *n* = 9	No Ir-GD *n* = 53	*P* value	All *n* = 62
**Age at EGD, mean (±SD)**	67 (15)	68 (13)	.992	68 (13)
**Female, *n* (%)**	3 (33)	24 (45)	.719	27 (44)
**Tumor origin, *n* (%)**			.410	
** NSCLC**	5 (56)	23 (43)		28 (45)
** Melanoma**	0 (0)	7 (13)		7 (11)
** HCC**	2 (22)	5 (9)		7 (11)
** Miscellaneous**	2 (22)	18 (34)		20 (32)
**Setting of treatment, *n* (%)**			.228	
** Metastatic or advanced- first line**	4 (44)	32 (60)		36 (58)
** Metastatic or advanced- subsequent line**	3 (33)	18 (34)		21 (34)
** nonmetastatic (ADJ, NA, maintenance)**	2 (22)	3 (6)		5 (8)
**ICI type, *n* (%)**			.511	
** Anti-PD-1 (pembrolizumab, nivoumab, cemiplimab)**	7 (78)	36 (68)		43 (70)
** Anti-PD-L1 (durvalumab, atezolizumab)**	2 (22)	10 (19)		12 (19)
** Anti-PD-1 with anti-CTLA-4**	0 (0)	7 (13)		7 (11)
**Combination therapy other than anti-PD-1 with anti-CTLA-4 within prior four months^a^, *n* (%)**	2 (22)	15 (28)	1.000	17 (27)
**Prior PPIs therapy, *n* (%)**	2 (22)	17 (32)	.709	19 (31)
**Prior corticosteroids use, *n* (%)**	0 (0)	15 (28)	.098	15 (24)
**Prior radiotherapy within four months, *n* (%)**	1 (11)	9 (17)	1.000	10 (16)
**Prior IBD or Celiac disease, *n* (%)**	3 (33)	2 (4)	**.019**	5 (8)
**Other ir-AEs, *n* (%)**	6 (67)	25 (47)	.473	31 (50)
**Ir-colitis**	1 (11)	4 (8)	.557	5 (8)
**Ir-pneumonitis**	3 (33)	4 (8)	.056	7 (11)
**Ir- thyroiditis**	2 (22)	8 (15)	.629	10 (16)
**Ir-AE other**	1 (11)	21 (40)	.140	22 (36)

a Including combinations with chemotherapy, mABs or TKIs.

Bold values represent statistically significant differences (P < 0.05).

Abbreviations: ADJ, adjuvant; CTLA-4, cytotoxic T-lymphocyte associated protein 4; EGD, esophagogastroduodenoscopy; HCC, hepatocellular carcinoma; IBD, Inflammatory bowel disease; ICI, immune checkpoint inhibitor; ir-AEs, immune-related Adverse Events; Ir-GD, immune-related gastritis or duodenitis; mABs, monoclonal antibodies; NA, neoadjuvant; NSAIDs, nonsteroidal anti-inflammatory drugs; NSCLC, non-small cell lung cancer; PD-1, programmed cell death protein 1; PD-L1, programmed cell death ligand 1; PPIs, proton-pump inhibitors; TKIs, tyrosine kinase inhibitors.

Baseline use of proton pump inhibitors (PPIs) was similar between patients with and without ir-GD (22% vs 32%, *P *= .709). Overall, 15 patients had a history of corticosteroid use. None of them were diagnosed with ir-GD (0% vs 28%, *P *= .098). There was also no significant difference in the rate of prior radiotherapy to adjacent organs (11% vs 17%, *P *= 1.000). Interestingly, preexisting inflammatory conditions of the GI tract, such as celiac disease or inflammatory bowel disease (IBD), were significantly associated with ir-GD (33% vs 4%, *P *= .019). No significant associations were observed between ir-GD and the incidence of other ir-AEs, including overall ir-AEs (67% vs 47%, *P *= .473), ir-colitis (11% vs 8%, *P *= .557), ir-pneumonitis (33% vs 8%, *P *= .056), or ir-thyroiditis (22% vs 15%, *P *= .629).

### Symptoms and other indications for EGD in patients with and without ir-GD

EGD indications, timing, and findings of patients with and without ir-GD are described in Table 3. There was no significant difference in the timing of EGD after ICI initiation between patients with and without ir-GD (mean 9.9 vs 8.6 months, *P *= .276). Among patients diagnosed with ir-GD, the timing of diagnosis ranged widely from one to 23 months (Table 4). The EGD indications were also not significantly different between the two groups. Most patients (*n* = 38, 61%) underwent EGD due to symptoms; other indications included evaluation for asymptomatic anemia (10%), abnormalities on computed tomography (CT) imaging (10%), and follow-up or screening EGD (19%).

**Table 3.** Clinical indications, timing and findings of esophagogastroduodenoscopy (EGD) in patients with and without immune-related gastritis or duodenitis (ir-GD).

	Ir-GD *n* = 9	No Ir-GD *n* = 53	*P* value	All *n* = 62
**Months from ICI initiation to EGD, mean (±SD)**	9.9 (8)	8.6 (9)	.276	8.8 (9)
**EGD indication^a^:**				
**Symptoms (excluding GIB)**	6 (67)	20 (38)	.148	26 (42)
**GIB**	3 (33)	9 (17)	.358	12 (19)
**Follow-up or screening^b^**	0 (0)	12 (23)	.185	12 (19)
**Anemia workup without GIB**	0 (0)	6 (11)	.580	6 (10)
**CT abnormality**	0 (0)	6 (11)	.580	6 (10)
**Symptoms, *n* (%)**				
**Any symptom**	9 (100)	29 (58)	**.009**	38 (61)
**More than one symptom**	5 (56)	8 (15)	**.015**	13 (21)
**Nausea and/or vomiting**	4 (44)	8 (15)	.062	12 (19)
**Epigastric pain**	3 (33)	10 (19)	.381	13 (21)
**GIB**	3 (33)	9 (17)	.357	12 (19)
**Dysphagia**	2 (22)	11 (21)	1.000	13 (21)
**Heartburn**	2 (22)	2 (4)	.097	4 (7)
**Weight loss**	1 (11)	2 (4)	.381	3 (5)
**Endoscopic findings^c^, *n* (%)**				
**Any gastritis or duodenitis**	8(100)	9 (17)	**<.001**	17 (28)
**Only gastritis**	5(63)	6 (11)	**.001**	12 (20)
**Only duodenitis**	2 (25)	2 (4)	.097	5 (8)
**both gastritis and duodenitis**	1 (13)	1 (2)	.271	1 (2)
**Microscopic findings^c^,**				
**Gastritis or duodenitis**	8 (100)	5 (9)	<.001	14 (23)
**malignancy**	0 (0)	2 (4)	1	2 (3)

a Two patients had more than one indication for EGD referral.

b Including screening EGDs, EGDs performed for cirrhosis surveillance, follow-up of prior EGD findings, and EGDs conducted in conjunction with colonoscopy.

c One patient with ir-GD did not have available data regarding EDG findings.

Bold values represent statistically significant differences (P < 0.05).

Abbreviations: EGD, esophagogastroduodenoscopy; GIB, gastrointestinal bleeding; ICI, immune checkpoint inhibitors.

**Table 4.** Characteristics of individual patients with immune-related gastritis or duodenitis (ir-GD) including clinical setting, diagnostic work-up, management and outcome.

#	Age/sex	Tumor origin, clinical setting and ICI type	IBD or Celiac history	Ir-G/ir-D	Ir-GD timing after ICI initiation (months)	Symptoms	EGD findings	Microscopic findings	G^a^	Ir-GD treatment	ICI treatment outcome	Disease status and OS (months)
**1**	83/M	Met lung ADC, first line pembrolizumab	none	Ir-D	3.7	GIB	Two duodenal ulcers: Forrest III and IIa in the bulb and second portion. Gastric mucosa—normal.	Duodenum without diagnostic abnormalities. Gastric mucosa showing mild chronic gastritis, HP negative.	3	PPIs	Discontinuation due to ir-pneumonitis, no rechallenge	DWD, 4
**2**	72/M	Advanced HCC, first line atezolizumab-bevacizumab	none	Ir-G	1.3	GIB	Erosive gastritis in the antrum. Prepyloric Forrest III ulcer.	Antral mucosa showing chronic-atrophic gastritis with intestinal metaplasia. HP negative.	3	PPIs	Discontinuation due to PD, switched to another ICI for another year after ir-G without symptoms relapse.	DWD, 32
**3**	42/M	Met colon carcinoma, third line pembrolizumab	Crohn’s disease	Ir-D	2.2	GIB	Two ulcers in the duodenal bulb. Gastric mucosa— normal.	Severe, erosive, ulcerated duodenitis. Granulation tissue. Chronic-active inflammatory infiltrate. HP Negative.	3	PPIs	Discontinuation due to PD	DWD, 1
**4**	46/F	Stage IIB TNBC, KN522 protocol, pembrolizumab maintenance	Celiac disease	Ir-GD	15.5	Epigastric pain, nausea	Severe duodenitis with a Forrest III ulcer in the bulb, involving 50% of circumference. Gastric mucosa—normal.	Chronic duodenitis with increased intraepithelial lymphocytes. Villous atrophy. Antral mucosa showing chronic-active gastritis. HP negative.	2	PPIs	Discontinuation due to ir-GD, rechallenge with minimal symptoms, completed the protocol.	AWOD, 15
**5**	62/M	Met lung ADC, first line pembrolizumab	none	Ir-G	12.4	Nausea, dysphagia	Complete sloughing of gastric mucosa with spontaneous bleeding. Erosive and erythematous defuse gastritis. Duodenal mucosa—normal.	Severe chronic-active and atrophic gastritis with increased intraepithelial lymphocytes, neutrophilic infiltration, and severe neuroendocrine cell hyperplasia. CMV and HP negative.	3	CS+PPIs	Discontinuation due to ir-G, no rechallenge with SD at 24 months	AWD, 24
**6**	81/M	stage III Lung SqCC, durvalumab maintenance	none	Ir-G	12.9	Epigastric pain, heartburn	Antral gastritis. Duodenal mucosa—normal.	Reactive Gastropathy.	2	Continued PPIs	Completed the protocol with ir-G diagnosis, remained NED at 42 months	AWOD, 42
**7**	68/M	Advanced HCC, fourth line nivolumab	none	Ir-G	18.5	Epigastric pain, heartburn, dysphagia nausea, weight loss	Diffused erosive and erythematous gastritis with contact bleeding. Duodenal mucosa—normal.	Severe chronic-active gastritis. HP negative.	3	CS+PPIs	Discontinuation due to ir-G, rechallenge without symptoms relapse.	AWD, 48
**8**	82/F	Met lung ADC, second line nivolumab	UC	Ir-G	23.3	vomiting	Esophagitis. Diffused erosive and edematous changes in the gastric mucosa. Duodenal mucosa—normal.	Chronic-active gastritis. Mild lymphocytic and neutrophilic infiltration. Foveolar hyperplasia, HP negative.	2	PPIs dose increase	Discontinuation due to ir-G, no rechallenge	DWD, 40
**9**	70/F	Met lung ADC, first line pembrolizumab	none	Ir-G	4.7	Epigastric pain, nausea and vomiting	Not available	Not available	3	CS+PPIs	Discontinuation due to ir-G, no rechallenge	DWD, 15

a ir-GD grading was defined using the Common Terminology Criteria for Adverse Events (CTCAE).

Abbreviations: ADC, adenocarcinoma; AWD, alive with disease; AWOD, alive without disease; CMV, cytomegalovirus; CS, corticosteroids; CTCAE, Common Terminology Criteria for Adverse Events; DWD, dead with disease; EGD, esophagogastroduodenoscopy; HP, *Helicobacter pylori*; HCC, hepatocellular carcinoma; IBD, inflammatory bowel disease; ICI, immune checkpoint inhibitors; ir-D, immune-related duodenitis; ir-G, immune-related gastritis; ir-GD, immune-related gastroduodenitis; KN522, keynote-522 (neoadjuvant paclitaxel, carboplatin, doxorubicin, cyclophosphamide and pembrolizumab followed by adjuvant pembrolizumab); Met, metastatic; NED, no evidence of disease; PD, progressive disease; PPIs, proton-pump inhibitors; SD, stable disease; SqCC, squamous cell carcinoma; TNBC, triple negative breast cancer; UC, ulcerative colitis.

All patients with ir-GD (*n* = 9, 100%) had at least one related GI symptom, compared to 29 of 53 (58%) without ir-GD (*P *= .009). Furthermore, multiple symptoms were more common among those with ir-GD (5 of 9 patients, 56%) than among those without ir-GD (8 of 53 patients, 15%; *P *= .015). However, no individual symptom occurred significantly more frequently in the ir-GD group, including epigastric pain (33% vs 19%, *P *= .381), nausea and vomiting (44% vs 15%, *P *= .062), GIB (33% vs 17%, *P *= .357), dysphagia (21% vs 22%, *P *= 1.000), heartburn, and weight loss.

### Detailed description of the cohort diagnosed with ir-GD

**Figure 1.**
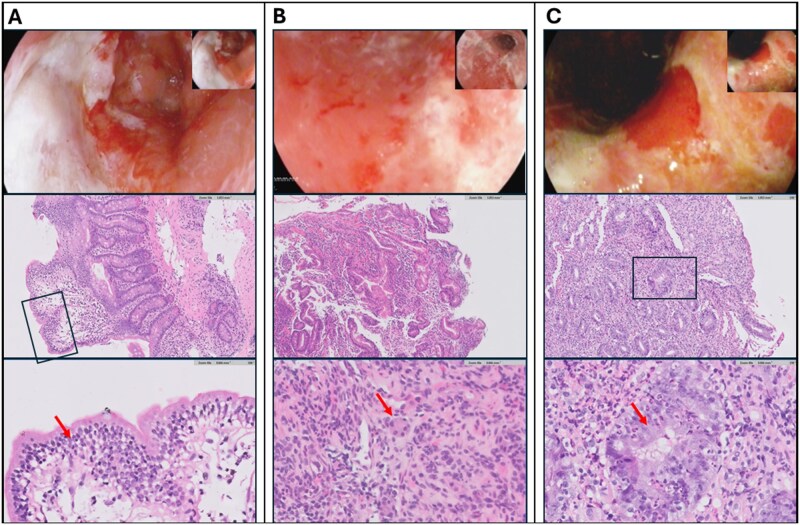
Endoscopic and Histopathological Findings of Specific Patients. (A) Patient #4: Severe duodenitis with a Forrest III ulcer in the bulb, involving 50% of circumference. Biopsy demonstrated chronic duodenitis and increased intraepithelial lymphocytes (arrow). (B) Patient #5: Sloughing of gastric mucosa with spontaneous bleeding. Gastric biopsy demonstrated chronic atrophic gastritis with marked neuroendocrine hyperplasia (arrow). (C) Patient #7: Erosive and erythematous gastritis with contact bleeding. Biopsy demonstrated severe chronic active gastritis, with infiltration and destruction of the gastric pits by neutrophils (arrow). The upper row shows representative pictures from esophagogastroduodenoscopy; the middle and lower rows show hematoxylin and eosin-stained sections at 10X and 40X magnification, respectively.

The characteristics of individual patients with ir-GD are described in Table 4. Overall, six patients with ir-GD had isolated gastritis (67%), two had isolated duodenitis (22%), and one experienced combined gastritis and duodenitis (11%). Three of nine patients with ir-GD (33%) had CTCAE Grade 2, and six (67%) had Grade 3. As mentioned above, three patients (33%) had preexisting immune dysregulation of the gastrointestinal tract, including Crohn’s disease, celiac disease, and ulcerative colitis. Their presentation was atypical for their baseline disease and was therefore attributed to ir-GD. For example, a female patient with known celiac disease experienced epigastric pain and nausea and was found to have gastritis and severe ulcerative duodenitis, despite adherence to a gluten-free diet. Her symptoms improved with PPIs treatment and ICI withholding but reappeared with milder manifestations after ICI rechallenge (Table 4 and Figure 1A).

Among the eight patients with ir-GD who underwent EGD at our medical center, seven (88%) demonstrated mucosal erosions or ulcerations on endoscopy. Histopathologic evaluation revealed multiple patterns, with some patients exhibiting more than one pattern. Chronic active GD, characterized by a mixed inflammatory infiltrate of lymphocytes and neutrophils, was identified in five patients (63%). Increased intraepithelial lymphocytes (IELs) were observed in two patients (25%), with mild-to-moderate severity. Chronic inactive GD and atrophic gastritis were also observed in two patients each (25%). In addition, neuroendocrine cell hyperplasia was noted in one patient (13%) (Table 4 and Figure 1).

No consistent correlation was identified between clinical presentation, endoscopic findings, and histopathologic features in the ir-GD cohort. GIB occurred in two of three patients with ir-D vs one of six with ir-G. Notably, two of the three patients who required corticosteroids had a neutrophilic infiltrate, suggesting a more active and severe phenotype. Nevertheless, a neutrophilic infiltrate was also observed in three patients who did not require corticosteroids. These observations should be interpreted cautiously, given the small sample size.

### Management and outcomes of patients with and without ir-GD

As noted above, ir-AE incidence was not significantly different between patients with and without ir-GD (Table 5). Treatment discontinuation due to ir-AE was more common among patients with ir-GD, with a borderline statistically significant difference (67% vs 28%, *P *= .051). In five out of six patients (83%) with ir-GD who discontinued ICI, the reason was ir-GD, and in one patient (17%), discontinuation was due to pneumonitis. The rate of hospitalization due to ir-AE was significantly higher in patients with ir-GD compared to those without ir-GD (67% vs 15%, *P *= .003).

**Table 5.** Treatment modifications and outcomes in patients with and without immune-related gastritis or duodenitis (ir-GD).

	Ir-GD (*n* = 9)	No ir-GD (*n* = 53)	*P* value	All (*n* = 62)
**ICI discontinuation due to any ir-AE, *n* (%)**	6 (67)	15 (28)	.051	21 (34)
**ICI discontinuation due to ir-GD, *n* (%)**	5 (56)	0 (0)	**<.001**	4 (6)
**Hospitalization due to any ir-AE, *n* (%)**	6 (67)	8 (15)	**.003**	14 (23)
**Hospitalization due to ir-GD, *n* (%)**	5 (56)	0 (0)	**<.001**	5 (8)
**PPIs administration, *n* (%)**	8 (89)	9 (17)	**<.001**	17 (27)
**Corticosteroids administration for any Ir-AE, *n* (%)**	4 (44)	19 (35)	.715	23 (37)
**Corticosteroids administration for ir-GD, *n* (%)**	3 (33)	0 (0)	**.002**	3 (5)
**Median Overall Survival (months)**	24	16	.384	16

Bold values represent statistically significant differences (P < 0.05).

Abbreviations: ICI, immune checkpoint inhibitors; ir-GD, immune-related gastritis or duodenitis; ir-AE, immune-related adverse event; PPIs, proton-pump inhibitors.

Treatment included PPIs in eight of nine patients with ir-GD (89%), with one additional patient continuing baseline PPIs therapy. Notably, only three patients with ir-GD required corticosteroids for ir-GD management. A significantly higher proportion of patients with ir-GD received PPIs therapy compared to those without ir-GD (89% vs 17%, *P *< .001), whereas the rate of corticosteroid treatment for ir-AEs did not differ significantly between the groups (44% vs 35%, *P *= .715).

Three of the nine patients with ir-GD underwent ICI rechallenge, one of whom received a different ICI type. All three experienced no or minimal recurrence of symptoms. The three patients who underwent ICI rechallenge remained clinically stable for prolonged periods and were reported alive at 15, 32, and 48 months. Additionally, one patient who did not undergo rechallenge remained stable without active oncologic treatment for two years (Table 4). Overall survival (OS) among ir-GD patients was variable, ranging from one to 48 months from the date of EGD, reflecting the heterogeneity of cancer types and treatment settings. No significant difference in overall survival (OS) was observed between patients with and without ir-GD (Table 3).

## Discussion

Our interest in ir-GD was sparked by challenging cases encountered at our medical center; One of the challenges was that oncologic patients receiving ICI therapy often had more than one potential trigger for gastro-duodenitis. Additionally, in some patients, there were discrepancies between endoscopic and microscopic findings, with only one modality supporting an inflammatory process. These discrepancies created clinical dilemmas in patient management. To address these challenges, we developed a practical multifactorial method for ir-GD diagnosis (Table 1). Our diagnostic framework, which integrates clinical and pathological features, a temporal relationship to ICI, and the exclusion of alternative causes, is consistent with the established, multifactorial approach for other ir-AE diagnoses.^16^ For example, the diagnosis of immune-mediated colitis is based on the combination of concordant clinical symptoms with characteristic endoscopic and histopathological findings.^13^^,^^16^ Likewise, guidelines for other ir-AEs, such as pneumonitis, hepatitis, and pancreatitis, emphasize the critical exclusion of common competing etiologies in oncology patients, including infection, malignant involvement, concomitant drugs, and relevant baseline conditions.^14–17^ Adopting a two-of-three-criteria approach provides a flexible diagnostic strategy that captures the heterogeneous presentations of ir-GD. Importantly, this framework permits diagnosis even when alternative etiologies remain plausible, when supported by clinical response to corticosteroids or recurrence on ICI rechallenge. Using these criteria, we identified ir-GD in a patient with preexisting celiac disease.

Consistent with prior studies, we found poor concordance between clinical, endoscopic, and histopathologic assessment.^5^^,^^6^ No specific feature reliably distinguished ir-GD from gastritis of other etiologies. Chronic active gastritis was the predominant pattern (Figure 1C), followed by lymphocytic gastritis with increased IELs (Figure 1A), as previously reported.^11^^,^^12^ Notably, one patient exhibited marked neuroendocrine hyperplasia, which to our knowledge has not been described in ir-GD (Figure 1B).

Ir-GD is a rare ir-AE. We identified an incidence rate of 0.4% in a cohort of 2553 cancer patients treated with ICI. Another 0.4% were diagnosed with GD unrelated to ICI. Previous reports on the incidence of isolated ir-gastritis range from 0.8% to 1.4%.^10^^,^^11^ Tang et al. reported an incidence of 0.9% for ir-GD among all patients treated with ICI, with duodenitis specifically occurring in 0.2% of cases.^18^ The lower incidence in our study may reflect the stricter diagnostic criteria applied.

In all patients with ir-GD, immunotherapy was held at symptom onset, and high-dose PPIs were either initiated or continued. Corticosteroids were reserved for the more severe cases. This regimen proved effective in all patients, including three patients who underwent successful ICI rechallenge. These observations suggest that corticosteroid treatment is not mandatory in mild ir-GD. Given the rarity of ir-GD and the absence of formal guidelines, the decision to rechallenge must be individualized.

The recurrence rate of ir-AEs following ICI rechallenge is approximately 30% with anti–PD-1/PD-L1 monotherapy, and higher with anti–CTLA-4 monotherapy or combination therapy. Notably, most ir-AE recurrences are low-grade, and some patients develop a different ir-AE upon rechallenge.^12^^,^^19^^,^^20^ The recurrence rate of ir-GD following ICI rechallenge remains unclear, with available data limited to case reports and small case series suggesting that rechallenge may be relatively safe.^21–25^ We advocate a cautious approach, generally considering ICI resumption only when alternative treatment options are limited and complete symptom resolution has been achieved.

The concept of immune privilege refers to anatomical sites, such as the brain and eye, where the immune function is tightly regulated to prevent tissue damage.^26^^,^^27^ The upper GI tract, as a major route of foreign antigen entry, similarly relies on mechanisms that promote tolerance.^28^ These include tolerogenic macrophages and dendritic cells, as well as regulatory T cells. A key manifestation of this tolerogenic environment is the chronic persistence of *Helicobacter pylori* within the gastric mucosa.^29^ The low incidence of ir-GD further supports the notion of immune privilege within the gastro-duodenal mucosa.

Our study is the first to compare patients with ir-GD to those undergoing EGD during ICI treatment who do not have ir-GD. Aside from increased prevalence of preexisting inflammatory dysregulation of the GI tract, such as celiac disease or IBD, among patients with ir-GD, no difference in baseline demographic or clinical characteristics was observed between ir-GD and non-ir-GD cohorts. Moreover, there was no difference between ir-GD and non-ir-GD patients in EGD indications, with EGDs performed primarily due to symptoms. Although ir-GD patients reported more GI symptoms overall, no specific symptom was significantly more frequent in this group. These observations suggest that ir-GD may present as a subtle GI toxicity syndrome that needs to be distinguished from non-ir-GD.

Involvement of the gastric and duodenal mucosa has been reported in 0.5% to 5% of patients with IBD.^30^^,^^31^ While endoscopic and histological manifestations can vary, the presence of epithelioid granuloma, aphthoid, or longitudinal ulcers or a bamboo-joint-like pattern on EGD is highly suggestive of IBD-related GD. None of these EGD features were observed in the patients included in this cohort. Patients with IBD are known to be at increased risk for ir-colitis, however, no data have been reported regarding an increased risk for ir-GD. Immune-related celiac disease has been documented and can either develop or flare in response to ICI treatment.^32^ The prevalence of preexisting IBD or celiac disease was not reported in two previous retrospective ir-GD trials^11^^,^^18^ and was used as an exclusion criterion in another study.^10^

Haryal et al. reported that 38 of 54 patients (70%) with ir-gastritis also had concurrent immune-related colitis (ir-colitis) or enteritis, including cases of duodenitis.^10^ Similarly, Tang et al. found that 21 of 41 patients (51%) exhibited both upper and lower GI tract injury.^18^ In both studies, microscopic inflammation observed on biopsy was considered sufficient to indicate organ involvement. In contrast, in our cohort, only one in nine patients diagnosed with ir-GD also had ir-colitis (11%). This discrepancy may reflect less frequent use of concurrent upper and lower GI endoscopy when lower GI symptoms are absent. Additionally, in our study, corticosteroids that are frequently used for the treatment of ir-AEs, including colitis, were considered a potential alternative etiology for GD, a factor not addressed in the aforementioned studies.

Our study has several limitations. It is a single-center trial with a modest sample size. Additionally, due to its retrospective design, the true incidence of ir-GD may be underrepresented. Data collection was limited to information documented in the electronic medical record, lacking the systematic evaluation typically conducted in prospective studies. Furthermore, we only included patients who underwent EGD during ICI treatment, which may lead to a referral bias and may exclude asymptomatic and symptomatic ir-GD cases that were not referred to endoscopy.

Altogether this study contributes to the growing evidence that ir-GD is a rare and manageable ir-AE. We suggest that clinicians should avoid the use of corticosteroids in clinically mild cases and show that ICI rechallenge can be safely attempted.

## Data Availability

The data underlying this article will be shared on reasonable request to the corresponding author.
