# Correction to: Rare occurrence of immunotherapy-related gastritis and duodenitis in a single tertiary medical center: a diagnostic challenge

**DOI:** 10.1093/oncolo/oyag280

**Published:** 2026-08-11

This is a correction to: Tal Etan, Iddo Bar-Yishay, Ariel Greenberg, Eli Brazowsky, Miriam R Brezis, Shlomit Strulov Shachar, Mor Miodovnik, Ido Wolf, Yasmin Leshem, Rare occurrence of immunotherapy-related gastritis and duodenitis in a single tertiary medical center: a diagnostic challenge, *The Oncologist*, Volume 30, Issue 11, November 2025, oyaf349, https://doi.org/10.1093/oncolo/oyaf349

In the originally published version of this manuscript, errors were inadvertently introduced into the author affiliations section.

Affiliation 2 has been corrected to read: “Gray Faculty of Medicine, Tel Aviv University, Tel Aviv 69978, Israel” instead of: “The Tel Aviv University Faculty of Medicine recently changed its name from the Sackler Faculty of Medicine to the Gray Faculty of Medicine, Tel Aviv 69978, Israel”.

Affiliation 3 has been corrected to read: “Department Gastroenterology and Hepatology, Tel-Aviv Sourasky Medical Center, Tel Aviv 6423906, Israel” instead of: “School of Medicine, Gray Faculty of Medical and Health Sciences, Tel-Aviv University, Tel- Aviv 6997801, Israel”.

The co-authors Iddo Bar-Yishay, Ariel Greenberg, and Eli Brazowsky have now been correctly assigned to affiliation 2 instead of affiliation 1.

